# Detection of *Leishmania* RNA Virus in *Leishmania* Parasites

**DOI:** 10.1371/journal.pntd.0002006

**Published:** 2013-01-10

**Authors:** Haroun Zangger, Catherine Ronet, Chantal Desponds, F. Matthew Kuhlmann, John Robinson, Mary-Anne Hartley, Florence Prevel, Patrik Castiglioni, Francine Pratlong, Patrick Bastien, Norbert Müller, Laurent Parmentier, Nancy Gore Saravia, Stephen M. Beverley, Nicolas Fasel

**Affiliations:** 1 Department of Biochemistry, University of Lausanne, Epalinges, Vaud, Switzerland; 2 Department of Medicine, Division of Infectious Diseases, Washington University School of Medicine, St. Louis, Missouri, United States of America; 3 Department of Molecular Microbiology, Washington University School of Medicine, St. Louis, Missouri, United States of America; 4 French National Reference Centre for Leishmaniases, Département de Parasitologie-Mycologie, CHRU de Montpellier, Montpellier, France; 5 University Montpellier 1, Faculty of Medicine, UMR CNRS 5290/IRD 224/UM1/UM2 “MIVEGEC”, Montpellier, France; 6 Institute of Parasitology, Vetsuisse Faculty Berne, University of Bern, Bern, Switzerland; 7 Department of Dermatology, Hôpitaux du Valais, Sierre, Valais, Switzerland; 8 Centro Internacional de Entrenamiento e Investigaciones Médicas (CIDEIM), Cali, Colombia; National Institute of Allergy and Infectious Diseases, United States of America

## Abstract

**Background:**

Patients suffering from cutaneous leishmaniasis (CL) caused by New World *Leishmania* (*Viannia*) species are at high risk of developing mucosal (ML) or disseminated cutaneous leishmaniasis (DCL). After the formation of a primary skin lesion at the site of the bite by a *Leishmania*-infected sand fly, the infection can disseminate to form secondary lesions. This metastatic phenotype causes significant morbidity and is often associated with a hyper-inflammatory immune response leading to the destruction of nasopharyngeal tissues in ML, and appearance of nodules or numerous ulcerated skin lesions in DCL. Recently, we connected this aggressive phenotype to the presence of *Leishmania* RNA virus (LRV) in strains of *L. guyanensis*, showing that LRV is responsible for elevated parasitaemia, destructive hyper-inflammation and an overall exacerbation of the disease. Further studies of this relationship and the distribution of LRVs in other *Leishmania* strains and species would benefit from improved methods of viral detection and quantitation, especially ones not dependent on prior knowledge of the viral sequence as LRVs show significant evolutionary divergence.

**Methodology/Principal Findings:**

This study reports various techniques, among which, the use of an anti-dsRNA monoclonal antibody (J2) stands out for its specific and quantitative recognition of dsRNA in a sequence-independent fashion. Applications of J2 include immunofluorescence, ELISA and dot blot: techniques complementing an arsenal of other detection tools, such as nucleic acid purification and quantitative real-time-PCR. We evaluate each method as well as demonstrate a successful LRV detection by the J2 antibody in several parasite strains, a freshly isolated patient sample and lesion biopsies of infected mice.

**Conclusions/Significance:**

We propose that refinements of these methods could be transferred to the field for use as a diagnostic tool in detecting the presence of LRV, and potentially assessing the LRV-related risk of complications in cutaneous leishmaniasis.

## Author Summary

The endosymbiosis of viruses in microbes is a well-described and prevalent environmental partnership, where viruses offer their cellular host incentives of fitness in exchange for the use of their metabolic machinery. We have recently exposed this as an important factor in certain metastatic leishmaniases of South America, where the nucleic acid of a virus residing within some *Leishmania* parasites acts as a potent innate immunogen causing a destructive inflammatory response, which worsens disease. *Leishmania* RNA Virus (LRV) exists within many species of *Leishmania* as a stable infection; these LRV positive strains have been found throughout South America in cutaneous leishmaniases that are often complicated by the occurrence of infectious metastasis with an underlying hyperinflammatory response. In this report, we describe the use of an anti-dsRNA monoclonal antibody (J2), which specifically recognizes dsRNA in a quantitative and sequence-independent fashion. Refined versions of these methods could be transferred to the field as diagnostic tools for detecting the presence of LRV (or other dsRNA viruses), and potentially assessing the LRV-related risk of complicated cutaneous leishmaniasis.

## Introduction

Leishmaniasis is one of the most important human protozoan parasitic diseases worldwide, with a prevalence of 12 million infections and a further 350 million people living at risk across 98 countries [1], [2]. It mainly presents in two major clinical forms: 1) cutaneous leishmaniasis (CL) in which lesions are generally localized and self-healing or 2) visceral leishmaniasis (VL) known to fatally disseminate to viscera. CL can be caused by various species, either from the *Leishmania (Leishmania)* subgenus (e.g. *L. major*, *L. mexicana* and *L. amazonensis*) or members of the *L. (Viannia) subgenus* (e.g. *L. braziliensis*, *L. panamensis* and *L. guyanensis*), while VL is mostly attributed to *L. donovani*, *L. infantum* and *L. chagasi*. Beyond the intrinsic parasite factors that seem to determine disease phenotype, extrinsic factors within the host are also known to alter the symptomatic spectrum of leishmaniasis [3].

In South America, CL patients mainly infected by *L. braziliensis*, *L. panamensis* and *L. guyanensis* are at risk for developing mucosal (ML) or disseminated cutaneous leishmaniasis (DCL) [3], [4], [5], [6], which are complications of CL involving dissemination of parasites from primary lesions to secondary sites, with or without mucosal involvement, and causing lesions that are often associated with a highly destructive inflammatory response [7], [8], [9], [10]. Mucosal disease is notorious for its poor response to commonly used treatments, such as antimony, and is often complicated by secondary bacterial or fungal infections. Very little is known about the pathogenesis of metastatic and mucosal leishmaniasis; especially the source of the uncontrolled inflammatory response observed in some patients. Two factors that have been associated with mucosal and disseminated disease include host genetic polymorphisms (e.g. in TNF, IL-6 and HLA genes) and HIV co-infection [11], [12], [13].

Recently, we suggested that the presence of a parasite dsRNA virus could contribute to the severity of the disease in strains of *L. guyanensis* [14], [15], [16]. This *Leishmania* dsRNA virus (LRV) has been found in various *L.* (*Viannia*) species as well as in one *L. major* strain [17]. Notably, in murine models of *L. guyanensis* infection, the LRV dsRNA genome is innately recognized by host Toll-like-receptor (TLR3), exacerbating the disease in a dose-dependent manner [14], [15].

*Leishmania* has a digenetic life cycle, with a motile extracellular promastigote form in the midgut of a female sand fly, and a non-motile intracellular amastigote form in the mammalian host macrophage. Our model proposes that the innate recognition of LRV takes place in the first few hours of infection. Here, some fraction of parasites die, releasing viral dsRNA that then binds to Toll-like receptor 3 (TLR3) trigging the subsequent IFN-type I driven inflammatory cascade that worsens disease [14], [18]. A high LRV burden in infecting parasites could therefore be a major determinant of disease severity and pathology.

LRV is a member of the *Totiviridae* family that regroups viruses found in several kingdoms of life [16], including protozoan parasites such as *Giardia*, *Trichomonas vaginalis*, fungi such as *Helminthosporium sp.* and *S. cerevisiae* as well as mosquitoes [19] and salmon [20]. They are small and simple virions (30–50 nm), containing a dsRNA genome that encodes its single capsid protein and an RNA-dependant RNA polymerase (RdRp), necessary and sufficient for both viral genomic dsRNA replication and viral ssRNA transcription. Viral transcripts are translated in the host cell cytoplasm into a capsid protein and, in most *Totiviridae*, into a fusion capsid-RdRp polypeptide (82 kDa and 176 kDa respectively). According to detailed studies in yeast, a single virion is composed of more than a hundred capsid protein molecules and one to two capsid-RdRp subunits surrounding the single genomic dsRNA molecule [21]. LRVs were identified and characterized several years ago in *L.* (*Viannia*) *braziliensis* and *guyanensis* [22], [23], [24] as well as in a single isolate of *L. major* [17]. Although their genomic organization is identical, high diversity in nucleotide sequence (less than 40% homology according to [17]) between LRVs of *L. (Viannia)* and *L. major* has categorized *Leishmania* viruses into the groups LRV1 and LRV2 respectively [17].

An important finding from our prior work is that only parasites with high levels of LRV exacerbated disease severity [14], [15], and previous studies have shown that considerable diversity in sequence is found amongst LRVs [17]. Studies looking into the role of LRV would thus be greatly aided by the availability of diverse methods for LRV detection and quantification, especially simple, rapid and reliable techniques, suitable for screening a large number of parasite strains in the field. To this end, we used parasite strains bearing different levels of LRV as standards [14]. Reliable detection and quantification was achieved by dsRNA extraction, quantitative real-time PCR (qRT-PCR) as well as with the immuno-detection of LRV genome in lysed, fixed or live parasite samples (ELISA, dot blot and fluorescence microscopy). Although qRT-PCR can be used efficiently and is a powerful method for detailed molecular studies on reference strains, it could have limited application for LRV screening on uncharacterized parasites from the field due to possible nucleotide and amino acid polymorphisms of LRVs. This problem was addressed by focusing on detection of dsRNA through the use of an anti-dsRNA monoclonal antibody (J2), which specifically recognizes dsRNA independent of its underlying nucleotide sequence. We applied this approach on several catalogued human isolates, on a fresh *L. braziliensis* sample obtained from a patient as well as on murine lesions biopsies, showing the relative ease of use of these methods for field application. We propose the technique as having great diagnostic potential for predicting the LRV-related risk of leishmanial dissemination.

## Methods

### Ethical statement

This study was approved by the Ethics Committee of Canton of Vaud, Lausanne (Switzerland) for the analysis of *Leishmania* parasites isolated from patients. The two *L. braziliensis* parasites strains (MHOM/BO/2011/2169 and MHOM/BO/2011/2192) were isolated from a *L. braziliensis* infected patient, who signed an informed consent accepting the use of materials for publication. Other *Leishmania* parasite strains used in this study are common lines isolated several years ago and described in several reports.

### Parasite strains and cultures

Different *L. guyanensis* reference strains of known LRV content [14] were used: i) two clones derived from the M4147 population (MHOM/BR/75/M4147) infected or not by LRV designated here as *Lg* M4147 LRV^high^ (M4147/*SSU:IRSAT-LUC(b)*) and *Lg* M4147 LRV^neg^ (M4147/pX63HXG/*SSU:IRSAT-LUC(b)*) respectively [25], ii) human isolates of *L. guyanensis Lg* 1398 (MHOM/BR/89/IM3597) and *Lg* 1881 (MHOM/BR/92/IM3862) and iii) *L. guyanensis* M5313 parasites (WHI/BR/78/M5313) and their derived non-metastatic (*Lg* 03 and *Lg* 17) or metastatic (*Lg* 13 and *Lg* 21) clones [14], [26]. Five human isolates of *L. braziliensis*, previously shown to be LRV-infected [24], were also analyzed: MHOM/CO/88/1407C (*Lb* 1407C), MHOM/CO/88/1407M (*Lb* 1407M), MHOM/CO/88/1403 (*Lb* 1403), MHOM/CO/86/1174 (*Lb* 1174) and MHOM/CO/84/1064 (*Lb* 1064). Two strains of *L. braziliensis* parasites were freshly isolated from an infected patient who contracted leishmaniasis: MHOM/BO/2011/2169 (from primary cutaneous lesion) and MHOM/BO/2011/2192 (from secondary/metastatic lesion), referred to in the text as *Lb* 2169 and *Lb* 2192.

Parasites were cultivated as promastigotes at 26°C in freshly prepared Schneider's insect medium (Sigma) supplemented with 10% heat-inactivated fetal bovine serum (PAA), 10 mM Hepes (Amimed), 50 U/ml penicillin/streptomycin (Amimed), 0.6 mg/L biopterin (Sigma) and 5 mg/L hemin (Sigma).

### Viral dsRNA extraction from total nucleic acids

Stationary phase *Leishmania* promastigotes were lysed for 20 min at RT with 0.4% sarkosyl and protease inhibitors (Roche) diluted in 1×PBS (10^8^ parasites in 100 µl). The lysates were then incubated at 37°C, first for 30 min with 400 µg/ml of recombinant proteinase K (Roche), then for a further 2 h with 10 µg/ml RNase (DNase-free from Roche). Nucleic acids, containing genomic parasitic DNA and LRV dsRNA, were extracted from these lysates by phenol-chloroform (at least twice), precipitated with 0.3 M sodium-acetate in 70% ethanol, then washed and resuspended in water (approx. 20 µl for 10^8^ parasites). DNA was quantified by spectrophotometry (Nanodrop). Pure viral dsRNA was obtained after RQ-DNase digestion according to manufacturer's instruction (Promega). Nucleic acids were analysed on 0.6% to 1.2% agarose gels containing SYBR-safe for nucleic acid staining (Invitrogen).

### Quantitative real-time PCR (qRT-PCR)

RNA was extracted from stationary phase promastigotes (approx. 3×10^7^) using Trizol (Invitrogen) according to manufacturer's instruction (1 ml Trizol for a 1 ml promastigote culture). After extraction, precipitation and washing, RNA was resuspended in water (3×10^7^ parasites in 10 µl) and quantified by spectrophotometry. 0.5–1 µg of RNA was then used for cDNA synthesis with SuperScript II Reverse Transcriptase (Invitrogen), which was finally purified with a QIAquick PCR purification kit (Qiagen). qRT-PCR was undertaken in a reaction solution of 0.5 µM primer diluted in SYBR Green Master mix (LightCycler 480 system, Roche). The reaction consisted of an initial denaturation at 95°C for 5 min followed by 40 cycles of amplification: 10 s at 95°C, 10 s at 60°C, 10 s at 72°C and a fluorescence detection step at 78°C to quantify the amplified DNA after each cycle. The following DNA oligonucleotides (Microsynth, Switzerland) were used: SetA: 5′-CTG ACT GGA CGG GGG GTA AT-3′ and 5′-CAA AAC ACT CCC TTA CGC-3′/SetB: 5′-GTC TGT TTC GTA CCC GCC G-3′ and 5′-AAG CTC AGG ATG TGC ATG TTC CA-3′/*kmp11* specific primers: 5′-GCC TGG ATG AGG AGT TCA ACA-3′ and 5′-GTG CTC CTT CAT CTC GGG-3′. SetA and SetB were based on LRV1-4 genome sequence (GenBank accession number: NC003601) and *L. major kmp11* gene as described previously [14]. LRV transcript levels were quantified in triplicate relative to the *Leishmania kmp11* housekeeping gene. Analysis and acquisition of data were performed with the LightCycler software 1.5 (Roche) using the 2^−ΔΔCT^ method.

### Anti-capsid antibody production and immunoblotting

The LRV capsid open reading frame was amplified from a cDNA preparation of *Lg* M5313 and cloned in a pET-28a *E. coli* expression vector (Merck). Its sequence was found to be highly similar to the capsid sequence of *Lg* M4147 LRV1-4 (more than 98% identical residues, Genbank accession number: JX313126). Recombinant capsid was purified, using a HIS-tag, then used for rabbit immunization (Covalab, polyclonal antibody identification code: g018d53). Proteins from total parasite extracts were quantified by BCA, 40 µg was loaded and separated on a 10% polyacrylamide denaturing gel, transferred to a nitrocellulose membrane and vizualised by Ponceau Red staining. After a 1 h blocking step in 5% powdered milk diluted in TBS+0.05% Tween20, the membrane was incubated overnight at 4°C with the g018d53 anti-capsid polyclonal antibody (1∶5000 in 1% milk TBS-Tween20). Following 4 washes of 15 min at RT, the membrane was incubated for 1 h with an anti-rabbit IgG antibody coupled to peroxidase (Promega) (1∶2500 in 1% milk TBS-Tween20), washed again 4× and finally revealed by ECL chemiluminescence (Amersham).

### Peptide arrays on cellulose membranes and epitope mapping

For antibody epitope screening, seventy-four 20-mer overlapping peptides (with an overlap of 10 residues) that cover the whole sequence of *Lg* M4147 LRV1-4 capsid (Genbank accession number: NC003601) were synthesized and attached to cellulose membranes by the Protein and Peptide Chemistry Facility (University of Lausanne).

The peptides were synthesized by using Intavis MultiPep synthesizer (Intavis Bioanalytical Instruments AG, Cologne, Germany). The cellulose membrane used was an Amino-PEG500-UC540 sheet (acid-hardened with improved stability). The membrane peptide linker was stable in wide range of aqueous pH (pH 0–pH 14) at ambient temperature for 12 hours. The PEG spacer consisted of 8–10 ethylene glycol units and had free terminal amino groups to start the peptide synthesis. The Amino- PEG500 spacer was loaded at 400 nmol/cm^2^ with a typical spot diameter of 4 mm and therefore an average of 50 nmol peptide/spot. The peptides were synthesized by stepwise solid phase synthesis. Amino acids that had N-terminal/side-chain protecting groups were spotted (if required) by robot. The amino acid solutions were activated using diisopropylcarbodiimide/hydroxybenzatriazole chemistry. For each cycle, solutions of the 20 common amino acids were dispensed along with solutions of modified amino acids as required (e.g. phosphorylated amino acids, acetylated amino acids, methylated amino acids). Following addition of the first amino acids, the membranes were treated to prepare the spots for the next in sequence. This was done by removing the N-terminal protective group (Fmoc) by piperidine. This cycle was repeated until the peptides reached the required length. Arrays were then treated with trifluoroacetic acid to reveal the native side chains. Arrays were stored at −20°C prior to use.

Similarly to the classic nitrocellulose membranes as described above, these peptide-spotted membranes were incubated with the g018d53 anti-capsid polyclonal antibody (1∶5000) to allow the determination of the epitopes for which it was specific.

### LRV sequencing

*Lg* 1398 LRV genome was partially sequenced as follows: first, viral dsRNA was obtained from approximately 10^9^ stationary phase promastigotes after total nucleic acids extraction and RQ-DNase digestion of genomic DNA (see “Viral dsRNA extraction from total nucleic acids” section) and purification of the 5.3 kb band after 0.8% agarose gel electrophoresis using Wizard SV gel and PCR clean-up system (Promega). Viral cDNA was then synthesized as described above (“Quantitative real-time PCR” section) and 10–50 ng was used for PCR amplification with 0.4 µl of GoTaq DNA polymerase (Promega) in its buffer supplemented with Q solution (Qiagen), 0.4 mM dNTPs (Promega) and 0.3 µM of each oligonucleotides (Microsynth, Switzerland). The PCR reactions consisted of 35 cycles: 1 min at 94°C, 1 min at 55°C and 2 min at 72°C. Two PCR fragments were generated and sequenced (by Fasteris, Switzerland) with the following oligonucleotides: i) 5′-GGA TCC GAA ACG TAA GCA AGT TTC TTG-3′ and 5′-CCA ATA CCA TGG CGC CAT CAC ATT CAT-3′ (based on LRV1-1 and 1-4 sequences) and ii) 5′-GAG AAA TAG CGA TAT CGC AGC CCA A-3′ (based on *Lg* 1398 LRV sequence obtained from previous reaction) and 5′-CAC AGC CAA CGT GAC GAC CAG AAA TCA C-3′ (LRV1-4). These two products allowed us to obtain 3.3 kb of *Lg* 1398 LRV genome sequence including the complete open reading frame of the viral capsid.

### Immunofluorescence microscopy (IFM)

Two different protocols were used. In protocol A, stationary phase promastigotes were fixed with 4% formaldehyde in PBS for 20–30 min (or overnight in 1% at 4°C), washed and resuspended at 2×10^7^ parasites/ml then attached to poly-lysine (Sigma) coated slides (Thermo Scientific) for 30 min at RT. After a 10 min permeabilization step in PBS+0.1% TritonX-100 (PBS-TX), cells were blocked for 45–60 min in 2% bovine serum albumin (BSA, Acros Organics) in PBS-TX, and incubated overnight at 4°C with the rabbit g018d53 anti-capsid polyclonal antibody (1∶5000) or the mouse anti-dsRNA J2 antibody (1∶800, English & Scientific Consulting) in 1% BSA in PBS-TX. Cells were then washed 4× in PBS, incubated for 1 h with a goat anti-rabbit IgG coupled to Alexa Fluor 594 (1∶2000, Invitrogen) or a goat anti-mouse antibody coupled to Alexa Fluor 488 (1∶600, Invitrogen) in 1% BSA in PBS-TX. These were washed twice, incubated 10–30 min with 0.5 µg/ml 4′,6-Diamidino-2-phenylindole (DAPI, Invitrogen), washed again and finally mounted with Vectashield diluted 100× in DABCO mounting solution (90% glycerol, 10% PBS and 2.5% DABCO from Sigma) or using Permafluor (ThermoScientific). Fluorescence visualization was performed with an Upright Axio Microscope at the Cellular Imaging Facility (CIF Epalinges, University of Lausanne).

In protocol B, 10^6^ parasites were fixed with 2% paraformaldehyde in PBS for 2 min. Cells were washed once in PBS and adhered to glass coverslips (Fisherbrand) by centrifugation (500 *g* for 2 min). Cells were permeabilized in blocking buffer (5% normal goat sera, 0.1% Triton-X100, 1× PBS) for 30 min at room temperature then incubated with mouse anti-dsRNA J2 antibody (1∶1000) for one hour. Cells were then washed 3× in PBS and incubated with goat anti-mouse IgG AlexaFluor 488 (1∶1000, Invitrogen) for one hour. After washing again in PBS (3×), coverslips were rinsed briefly in water and mounted using Prolong Gold (Invitrogen). Microsocopy was performed using Olympus AX70 microscope and images were obtained using QCapturePro software (Version 5.1.1.14). Image analysis was performed using Image J (1.45).

### Slot blot

5×10^6^ parasites were resuspended at a final concentration of 5×10^5^ cells/ml in PBS. 100 µl were adhered to nitrocellulose membranes using Mini-fold II Slot-Blot System (Schleicher & Schuell, Keane, NH). The membrane was incubated in 2% powdered non-fat milk for 1 h, then with mouse anti-dsRNA J2 antibody (1∶2000) and polyclonal rabbit anti-histone H2A (1∶2000; Wong and Beverley, in preparation) in 2% milk plus 0.2% Tween 20 for 1 h. The membrane was washed in 1×PBS plus 0.1% Tween 20 (PBS-T) 3× and incubated in goat anti-mouse IRDye 800 and goat anti-rabbit IRDye 680 (1∶10000 each, Licor Biosciences, Lincoln, NE) for 1 h. The membrane was washed 3× in PBS-T and once in 1×PBS. Analysis was performed using the Odyssey Infrared Imaging System and Application Software Version 3.0.16 (LiCor Biosciences). The cut-off point was calculated as 3 standard deviations (S.D.) above the mean absorbance of the LRV negative control.

### ELISA

Stationary phase promastigotes (10^8^ parasites/ml) were lysed in PBS+0.5% Nonidet P40 (NP40). 20 µg of total proteins, equating to approx. 5×10^6^ parasites (as quantified with BCA assay) were adhered to a 96 well plate (Thermo Scientific), which had been pre-coated with poly-lysine (Sigma), overnight at 4°C. After 4 washes in PBS 0.05% Tween20 (PBS-Tw20), lysates were blocked in assay diluent solution (eBioscience) for 2 h at RT, washed again in PBS-Tw20, and incubated for 1 h at 37°C with the primary mouse monoclonal anti-dsRNA J2 antibody (1∶2000, English & Scientific Consulting). After 4 more washing steps, a secondary anti-mouse IgG HRP conjugated antibody (1∶2500, Promega) was added for 1 h at 37°C. Wells were then washed and dsRNA could be colorimetrically quantified by the addition of o-Phenylenediamine dihydrochloride (OPD) in a phosphate citrate buffer (Sigma). The reaction was stopped by acidification with 0.5 M H_2_SO_4_ and measured at 490 nm with a Biotek Synergy HT spectrophotometer. The cut-off point was calculated as 3 standard deviations (S.D.) above the mean absorbance of the LRV negative control.

### Dot blot

Stationary phase promastigote pellets were resuspended in 1×PBS, and a small amount was lysed for BCA quantification in 0.5% NP40. Parasite samples in PBS were then adjusted to 0.1 µg/µl of total protein and spotted onto a nitrocellulose membrane using a range of 0.5 to 4 µg of protein per spot (corresponding to approx. 10^5^ to 10^6^ parasites). To test the sensitivity of the method, live parasites were counted, serially diluted between a range of 10 to 1000 parasites and directly spotted on the nitrocellulose membrane. The membranes were then dried before revelation by immunodetection as described above (see “Anti-capsid antibody production and immunoblotting” section), using an anti-dsRNA J2 primary antibody (1∶1000, English & Scientific Consulting) and an anti-mouse IgG HRP conjugated secondary antibody (1∶2500, Promega).

### Mouse infection and RNA extraction from leishmaniasis lesions

One million stationary phase *Lg* M4147 LRV^high^ or *Lg* M4147 LRV^neg^ promastigotes were injected subcutaneously into the base of the hind footpad of C57BL/6 mice. Lesions were isolated at the peak of infection (approx. 4 weeks post-infection) and homogenized with a mortar and a pestle in PBS. After an initial centrifugation step to remove large debris (50 g for 2 min), cell supernatant was centrifuged again (600 g for 8 min) and the pellet was directly resuspended in Trizol for total RNA extraction (as described in “qRT-PCR” section). Approximately 50 µg of RNA was obtained from each lesion (40–50 mg) and diluted in water for dot blot analysis with the J2 antibody (see “Dot blot” section).

## Results

In order to characterize the presence and burden of LRV in *L. (Viannia)* parasite strains *via* different methods, we first tested four parasite isolates of varying LRV content [14]. Two clones derived from the *L. guyanensis* M4147 strain were used: *Lg* M4147 LRV^high^, known to have a high burden of LRV and *Lg* M4147 LRV^neg^ in which LRV is undetectable by RT-PCR tests [25]. In addition, we also tested two human isolates of *L. guyanensis*: *Lg* 1398, derived from a metastatic lesion and known to bear high levels of LRV and *Lg* 1881, from a CL patient and in which LRV is present at a very low level (at least 10'000 fold less [14]). To best compare the various LRV detection techniques, each was performed on material from a single sample preparation (except for the slot blot). The data shown are representative of the trend gleaned from several independent experiments.

### LRV detection by gel electrophoresis and quantitative real-time PCR

**Figure 1:**
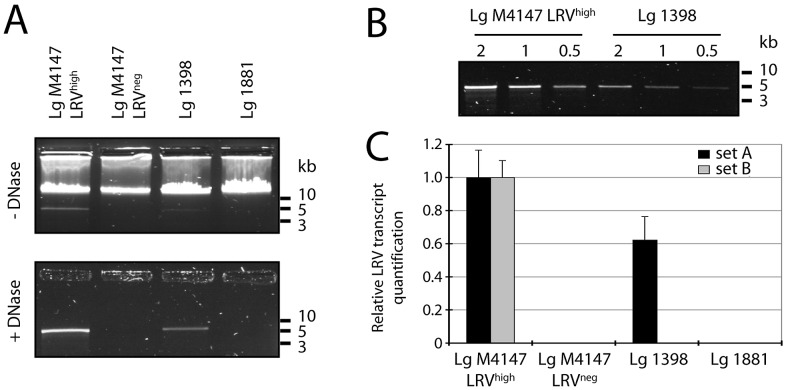
Detection of LRV in nucleic acid extracts. **A and B.** **Visualization of viral genomic dsRNA by gel electrophoresis.** **A.** Total nucleic acid from stationary phase promastigotes was treated with ssRNase then migrated in a 1% agarose gel. The sample was either kept intact (1 µg, upper panel) or digested with RQ-DNase (5 µg, lower panel). **B.** To quantify viral dsRNA in *Lg* 1398 relative to *Lg* M4147 LRV^high^, various concentrations of nucleic acid (2, 1 and 0.5 µg) were digested with RQ-DNase and migrated as above. **C.** **Quantification of LRV transcript by qRT-PCR.** Total parasitic and viral cDNA was prepared for qRT-PCR and amplified using primers specific for LRV (SetA and SetB, see material and methods for sequences). Viral transcript was quantified as normalized to the parasitic housekeeping gene *kmp11* then adjusted relative to *Lg* M4147 LRV^high^.

As a starting point, LRV content was estimated using two previously used methods [14]. Firstly, total nucleic acids were extracted from promastigote cultures and analyzed by agarose gel electrophoresis. Here, a 5.3 kb band corresponding to the size of the viral dsRNA genome was detectable in *Lg* M4147 LRV^high^ and *Lg* 1398 extracts, which was weaker in the latter (Figure 1A, upper panel). This band could be seen more clearly when parasite genomic DNA was eliminated by DNase treatment (Figure 1A, lower panel). As expected, LRV dsRNA was not detectable in *Lg* M4147 LRV^neg^ or in the LRV^low^ strain *Lg* 1881. Using a serial dilution of nucleic acids from LRV-infected parasites, we estimated that the amount of LRV dsRNA was approximately three to four times higher in *Lg* M4147 LRV^high^ than in *Lg* 1398 (Figure 1B).

We then quantified LRV transcript levels, after RNA extraction followed by cDNA synthesis, using two different primer sets that we have already successfully used for LRV detection in *Lg* M4147 and *Lg* M5313 strains and their clonal derivatives: SetA, which amplified a 124 nucleotide fragment on the 5′-end of the viral RNA (nucleotide 153 to 277 of the LRV1-4 sequence) [14], and SetB, which amplified a 103-nucleotide fragment in the *RdRp* open reading frame (nucleotide 3591 to 3694 of LRV1-4). Quantitative RT-PCR was performed and normalized to both the amplification obtained from the conserved *kmp11* housekeeping gene and the signal obtained from *Lg* M4147 LRV^high^. With the SetA primers, *Lg* 1398 showed nearly half the LRV transcripts than *Lg* M4147 LRV^high^, while the *Lg* M4147 LRV^neg^ line and *Lg* 1881 showed no detectable LRV product. Notable is that no product was obtained with the SetB primers from *Lg* 1398 despite having high levels of LRV (Figure 1B vs Figure 1C). Preliminary sequencing data of the *Lg* 1398 LRV *RdRp* open reading frame explained this negative result (H. Zangger, unpublished data), and illustrated a potential problem of a PCR-based approach for LRV screening in uncharacterized parasites.

### LRV detection by a capsid-specific antibody

**Figure 2:**
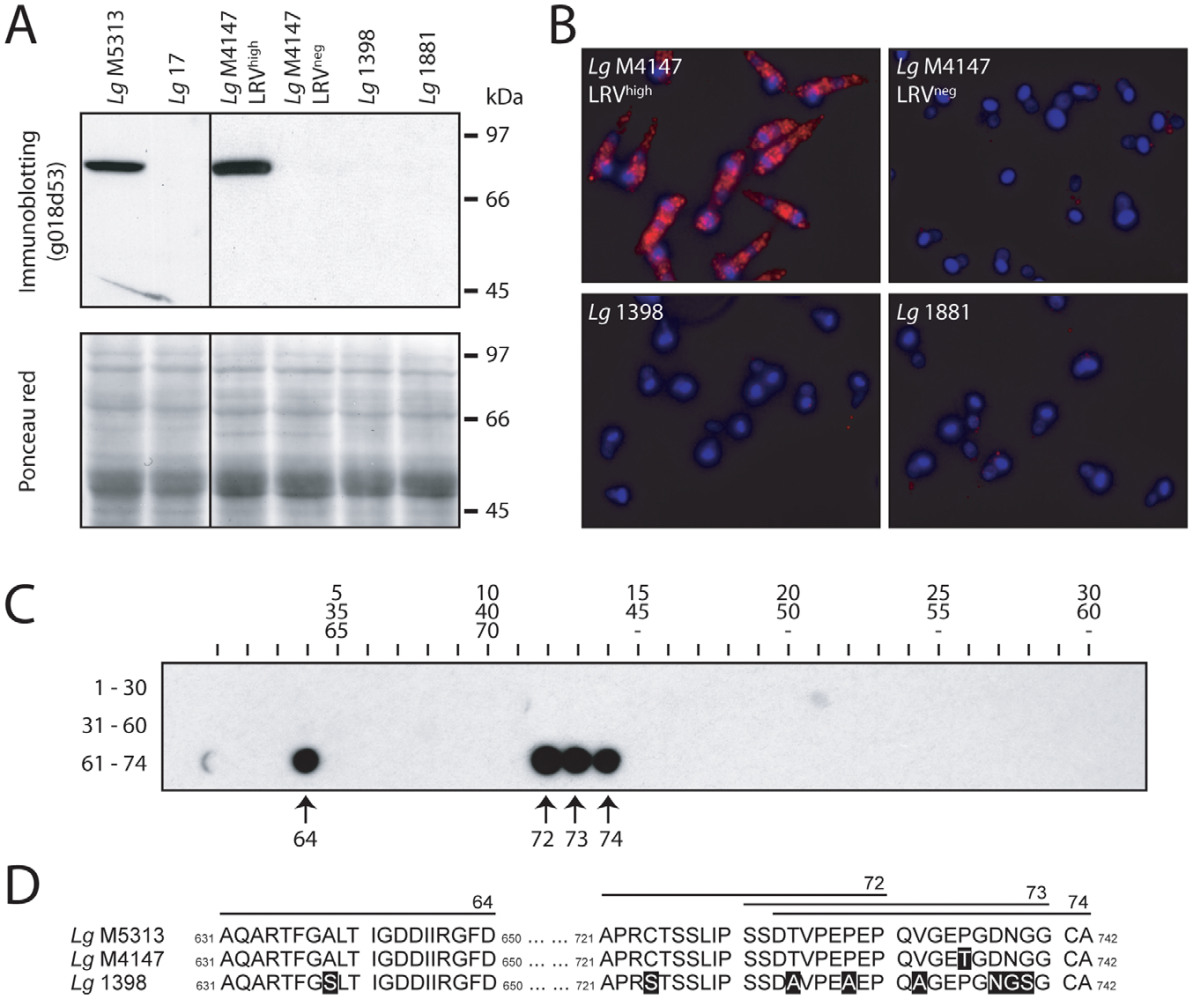
Detection of LRV with a polyclonal anti-capsid antibody (g018d53) and epitope mapping. **A.** **Western blot.** Total parasitic protein extract (40 µg) was separated on a 10% acrylamide denaturing gel then transferred onto a nitrocellulose membrane where the LRV capsid could be detected using the rabbit polyclonal antibody g018d53 (upper panel). A Ponceau staining of the same membrane shows total parasitic protein (lower panel). **B.** **Immunofluorescence microscopy.** Red: capsid (g018d53 Ab). Blue: DAPI integrated into kinetoplast and nuclear DNA. Capsid immunofluorescence was visualized with a standardized exposure time in all images. **C.** 74 overlapping peptides (20-mer) covering the complete sequence of *Lg* M4147 LRV1-4 capsid were spotted on a cellulose membrane (30 peptides per lane as indicated) and incubated with the g018d53 antibody to identify the recognized epitopes. **D.** Sequence alignment of the LRV capsids from *Lg* M4147, *Lg* M5313 and *Lg* 1398 in the C-terminal region covering the epitopes recognized by the g018d53 antibody (shown in C). The residues that are not identical to the *Lg* M5313 LRV sequence are highlighted in a black box.

Detection of LRV can also be performed *via* the recognition of viral proteins [27]. A high-affinity rabbit polyclonal antibody (g018D53) was raised against the capsid polypeptide of *Lg* M5313 LRV (>98% identical to *Lg* M4147 LRV1-4, Genbank accession number: JX313126) and then tested on control strains by immunoblotting and fluorescence microscopy. With both techniques, LRV detection was achieved in *Lg* M5313 (and its derivative LRV^high^ clones, *Lg* 13 and *Lg* 21; data not shown) as well as in *Lg* M4147 LRV^high^, showing a strong staining throughout most of the cytosol of promastigotes (Figures 2A and 2B). As expected, no staining was visible in *Lg* 17 (LRV^low^ derivative clone of *Lg* M5313), *Lg* M4147 LRV^neg^ and *Lg* 1881, but neither in the LRV-infected human isolate *Lg* 1398 which is probably due to LRV sequence diversity. Partial *Lg* 1398 LRV sequencing was performed and surprisingly revealed a high identity of its capsid as compared to *Lg* M4147 throughout the entire open reading frame (91% identical residues, Genbank accession number: JX313127). Epitopes mapping using a 20-mer peptide arrays representing the complete *Lg* M4147 LRV capsid sequence showed that g018D53 recognized uniquely *Lg* M5313 LRV C-terminal capsid sequence, which is poorly conserved in *Lg* 1398, thus explaining why it is not recognized by g018D53 in this strain (Figure 2C and 2D).

### Immunodetection of LRV by a dsRNA-specific antibody

**Figure 3:**
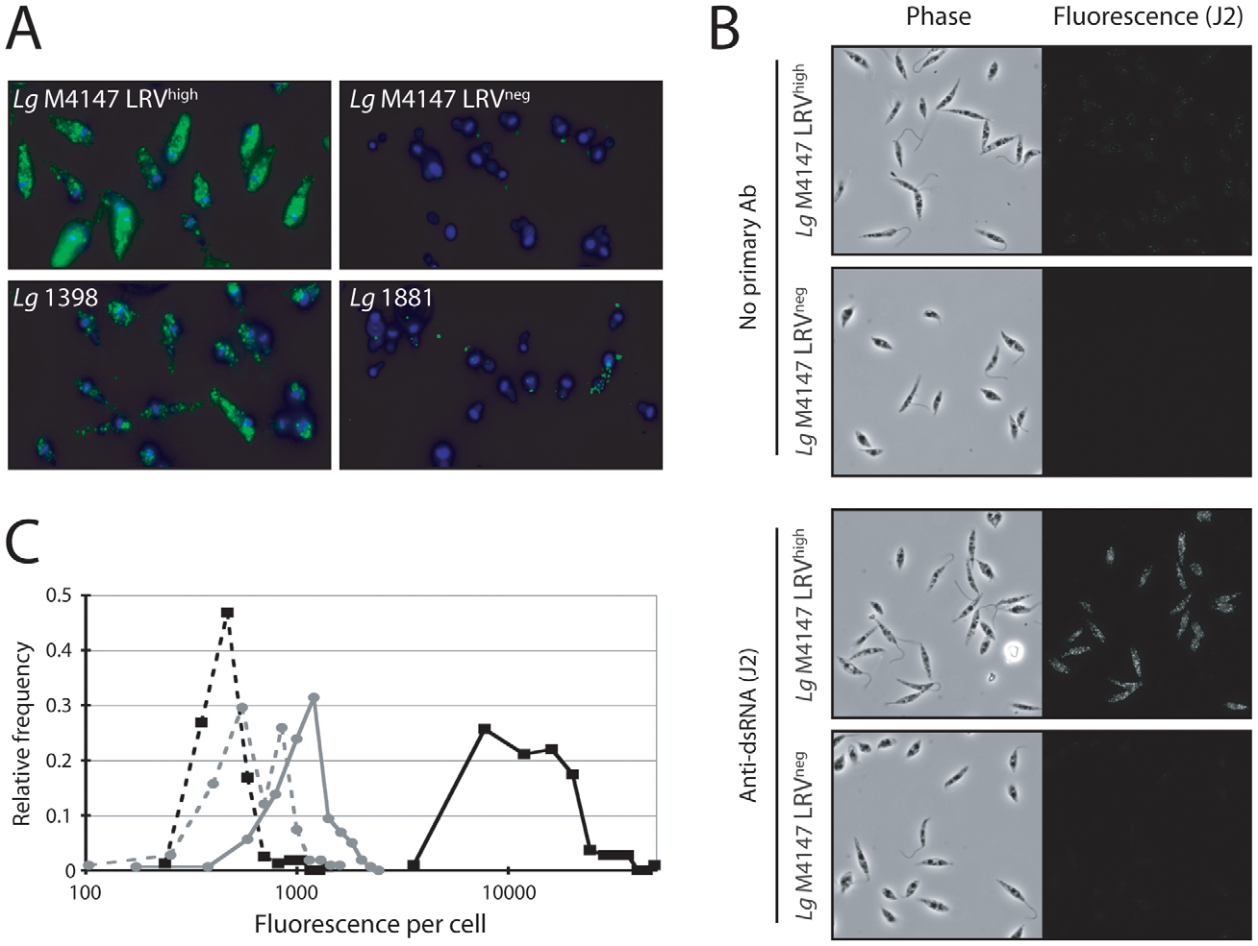
Detection of LRV with a monocolonal anti-dsRNA (J2) antibody by immunofluorescence microscopy. **A.** Reference strain analysis (protocol A, see “Material and methods”). Green: dsRNA (J2 Ab). Blue: DAPI (standardized exposure time in all images). **B.** Phase and immunofluorescent images of *Lg* M4147 LRV^high^ or LRV^neg^ cells were obtained in the presence or absence of J2 antibody (protocol B). **C.** Quantitative immunofluorescence (protocol B). The fluorescent intensity per cell was assessed using Image J software on *Lg* M4147 LRV^high^ or LRV^neg^ cells following IFM with the J2 antibody. Cells from phase images were identified and the fluorescent intensity average over the area of the cell was recorded. 108–160 cells from 2 distinct fields were measured, and histogram plots were made using Excel software. LRV^high^, no primary antibody (▪, dashed line); LRV^high^ with J2 (▪, solid line); LRV^neg^, no primary antibody (•, dashed line); LRV^neg^ with J2 (•, solid line).

The J2 monoclonal mouse antibody directed against dsRNA allows the detection of various dsRNA viruses independently of their sequences [28], [29]. To gauge its utility for LRV detection, it was first tested on control parasites by fluorescent microscopy using two different fixation protocols (Figures 3A and 3B). For both protocols the staining pattern with the J2 antibody was similar to that seen with the anti-capsid antibody (Figure 2B). Interestingly, a signal was obtained with the strain *Lg* 1398, suggesting that the anti-dsRNA antibody was not limited by differences in sequence amongst LRVs as noted earlier in the qRT-PCR and anti-capsid studies.

From the images acquired via the second protocol (Figure 3B), histograms were constructed to show the distribution of signal intensity between individual cells (Figure 3C). A distinct peak was seen in the *Lg* M4147 LRV^high^ line that was quite separated from that obtained with the LRV^neg^ line or controls (Figure 3C). The spread of the *Lg* M4147 LRV^high^ peak was somewhat broader than might have been anticipated for a homogeneous population, suggesting some heterogeneity in LRV levels may exist. Similar results have been obtained with anti-capsid antisera (FMK and SMB, not shown).

**Figure 4:**
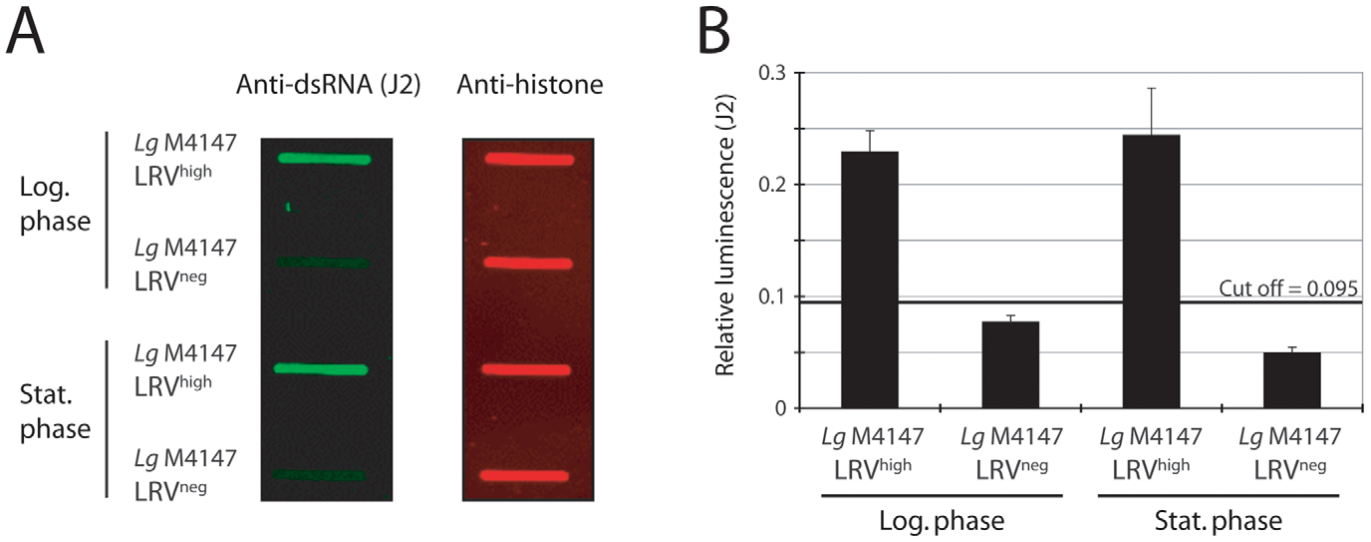
Detection of LRV using slot blots and J2 antibody. **A.** 5×10^4^ parasites were blotted onto nitrocellulose membranes and incubated with J2 or anti-histone H2A antibodies. **B.** Quantification of the signal intensity for cells in logarithmic or stationary growth phase: dsRNA signal was quantified relative to the histone H2A signal. The cut-off line was calculated as 3 standard deviations (SD) above the mean absorbance of the LRV-negative that showed the highest value (log phase).

We also tested the use of a slot-blot technique for estimating LRV load. In this protocol, cells were ‘slotted’ onto nitrocellulose membranes and reacted with J2 to detect dsRNA and anti-histone H2A to control for parasite numbers. Clear differences in LRV^high^ and LRV^neg^ parasites were again observed (Figures 4A and 4B). Both logarithmic and stationary cells were tested showing that the dsRNA signal intensity does not significantly change during culture of the parasite.

**Figure 5:**
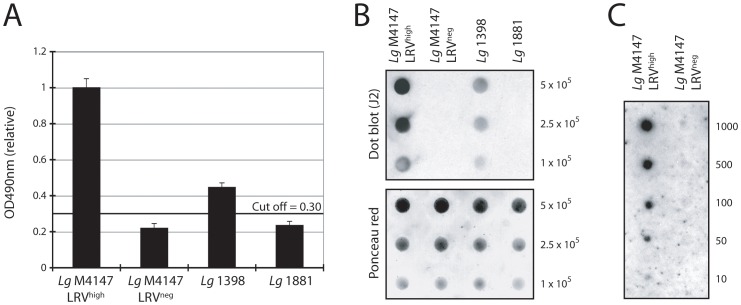
Detection of LRV in total parasite lysate using J2 antibody. **A.** **ELISA.** Total lysates from 5×10^6^ promastigotes were coated on 96 wells plates and dsRNA was quantified colorimetrically at 490 nm relative to *Lg* M4147 LRV^high^ after background subtraction (uncoated control wells). The cut-off line was calculated as 3 standard deviations (SD) above the mean absorbance of the LRV-negative that showed the highest value (*Lg* 1881). **B.** **Dot blot.** 10^5^ to 5×10^5^ promastigotes were spread directly onto a nitrocellulose membrane and dsRNA was detected using the J2 antibody (upper panel). A Ponceau stain of the membrane shows total protein concentration was similar across samples (lower panel). **C.** **Dot blot sensitivity screening.** A dot blot was performed in a serial dilution of 1000 to 10 parasites from LRV-positive and negative control strains (*Lg* M4147 LRV^high^ and *Lg* M4147 LRV^neg^).

The results obtained in IFM or ‘slot’ blotting prompted us to explore more rapid and simple protocols for the use of the J2 anti-dsRNA antibody that may be suitable for screening of field isolates, where sequence divergence amongst LRVs is expected. It was thus transferred to the other immunodetection techniques of ELISA and dot blot. The J2 ELISA method used crude parasite lysate (NP40); it allowed relative quantitation of LRV and confirmation that it was approximately four times more abundant in *Lg* M4147 LRV^high^ than in *Lg* 1398 (Figure 5A). However a clear limitation of this approach is the requirement for high LRV load as illustrated here with a relatively low signal obtained with the *Lg* 1398 strain in comparison to LRV-low/negative strains.

Dot blot tests were performed with whole live parasites spotted directly on nitrocellulose membranes. Distinction between infected or non-infected promastigotes was remarkably reliable (Figure 5B), permitting a relative quantification that reproduced the difference in LRV load between *Lg* M4147 LRV^high^ and *Lg* 1398 (Figures 1B and 5A). In addition to being a simple technique that is independent of LRV sequence, the dot blot had the advantage of only requiring a very low number of parasites as shown in Figure 5C. Here, LRV could be detected in less than a hundred parasites from the *Lg* M4147 LRV^high^ line.

### Screening for LRV infection in human isolates

**Figure 6:**
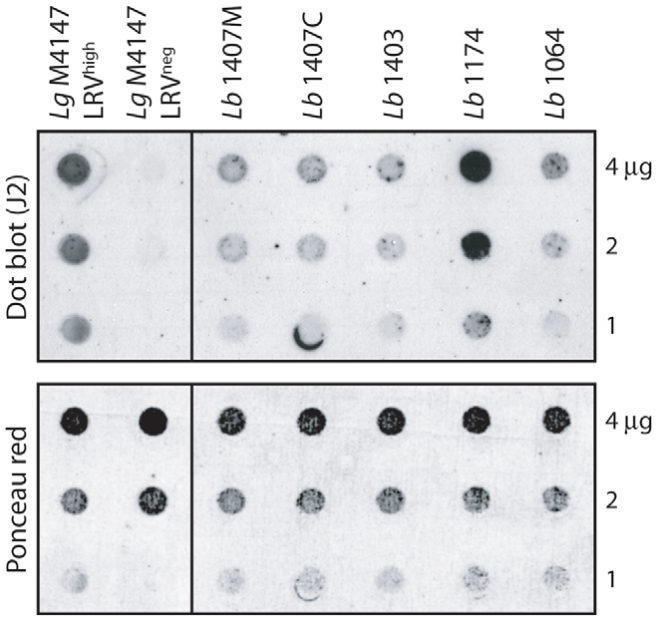
Screening for LRV in human isolates of *Leishmania*. Parasites of 5 different *L. braziliensis* strains previously shown to harbor LRV [24] were analyzed by dot blot (1 to 4 µg total protein/spot).

To assess the applicability of our anti-dsRNA dot blot on field isolates, we used it for LRV screening in human isolates from another *Leishmania* species that had been previously typed and catalogued as LRV positive [24]. Five strains were screened, corresponding to *L. braziliensis* isolated from human lesions (Figure 6). As expected, we were able to confirm LRV presence in these isolates. This study suggested that the dot blot method using J2 was a valid approach that can be extended to clinical *Leishmania* isolates from human biopsy.

### Screening for LRV presence in *L. braziliensis* isolated from an infected patient

**Figure 7:**
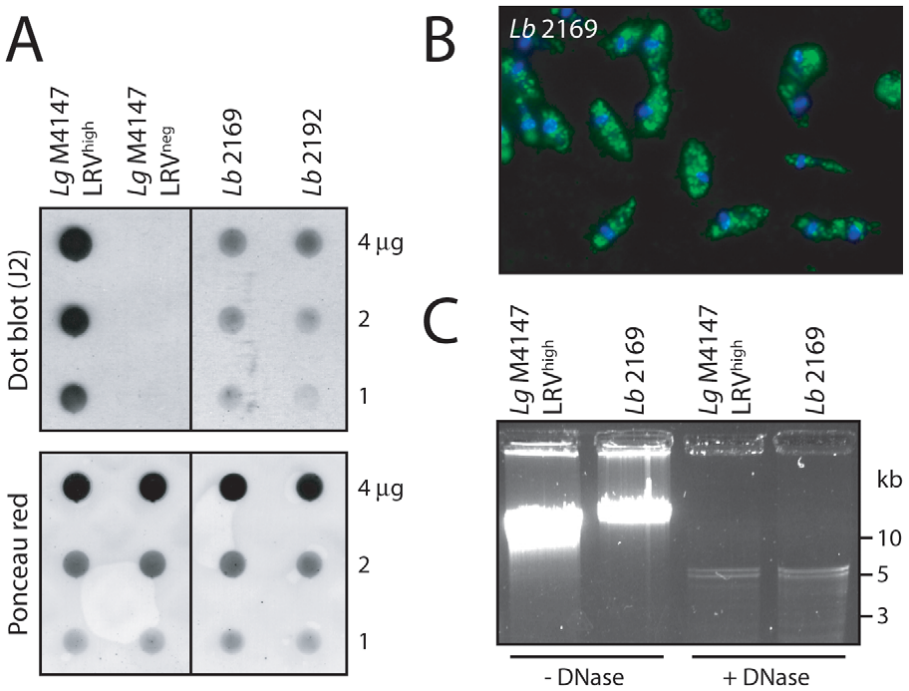
Screening for LRV in freshly-isolated human *L. braziliensis*. **A.** Dot blot analysis of two parasite samples obtained from separate lesion biopsies in an infected patient: *Lb* 2169 and *Lb* 2192. Live parasites (1 to 4 µg total proteins) were spotted on a nitrocellulose membrane for LRV dsRNA detection by dot blot (J2 antibody). *Lg* M4147 LRV^high^ and LRV^neg^ were used as positive and negative controls. Upper panel: dsRNA detection by dot blot (J2). Lower panel: verification of protein quantity by Ponceau staining. **B.** J2 anti-dsRNA analysis of *Lb* 2169 by fluorescence microscopy. Green: dsRNA (J2 Ab). Blue: DAPI. **C.** Isolation of viral genomic dsRNA from the *Lb* 2169 strain. Intact and DNase-digested total nucleic acids from *Lb* 2169 parasites and *Lg* M4147 LRV^high^ as a control, were analyzed by gel electrophoresis (similarly to Figure 1A). Note: with high resolution gels such as presented here (in contrast to Figure 1), the viral genome often appears as a doublet.

To demonstrate that our anti-dsRNA immunodetection approach may be a relevant diagnostic tool in a clinical setting, it was tested on freshly isolated *Leishmania* parasites obtained from an *L. braziliensis* infected patient. The subject contracted leishmaniasis in Bolivia, which was later typed by PCR as being *L. braziliensis* (data not shown). Two parasite samples were taken: *Lb* 2169 and *Lb* 2192, derived respectively from a primary cutaneous lesion before treatment, and a secondary/metastatic lesion appearing some time after treatment had started. Parasites from these biopsies were cultivated and directly tested for LRV presence by dot blot using the anti-dsRNA antibody as described above. *Lg* M4147 LRV^high^ and *Lg* M4147 LRV^neg^ parasites were used as positive and negative controls respectively. As shown in Figure 7A, a clear signal, although weaker than for *Lg* M4147 LRV^high^, was detected in both parasite isolates from this infected patient. To ascertain that this positive signal was genuinely due to the presence of LRV, the samples were retested using some of the other LRV detection techniques, i.e. immunofluorescence microscopy (Figure 7B) and isolation of viral dsRNA, with clear detection of a ssRNase- and DNase-resistant 5.3 kb band (Figure 7C). Sequencing of this newly identified LRV is currently in progress. Because the presence of LRV may be an aggravating factor in the development of refractory metastatic disease, early diagnosis of LRV content may aid diagnosis and be used to guide treatment strategies. This experiment demonstrated the ease and accuracy of dsRNA detection and reinforced the broad applicability of the anti-dsRNA antibody in the detection of LRV across *Leishmania* species.

### Screening of LRV in footpad lesions

**Figure 8:**
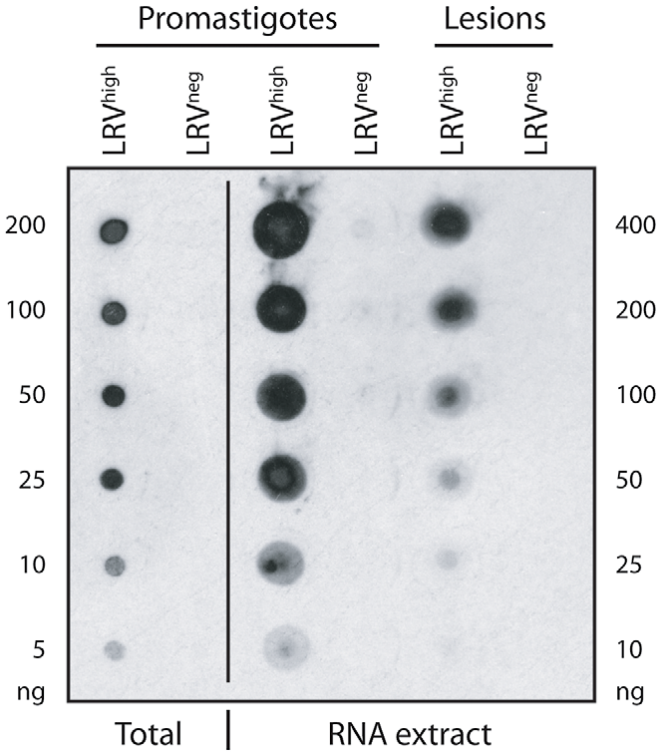
Detection of LRV in mice footpad lesions. Dot blot analysis on total RNA extracted from mice lesions infected with *Lg* M4147 LRV^high^ and *Lg* M4147 LRV^neg^. Whole parasite (‘total’) and RNA extracts from *Lg* M4147 promastigotes were also loaded as a control. The amount of protein and RNA loaded is indicated on the left and right side of the figure respectively.

Although these results suggest that our J2 antibody might have the potential to detect LRV for clinical application, a major limitation could be the need for parasite isolation from biopsy lesions, which is not always easy to achieve in the field. It was therefore important to test its direct applicability from lesions, thus avoiding parasite cultivation. A proof-of-concept study was performed on murine leishmanial lesions. After a simple RNA extraction with Trizol from leishmanial lesions of mice infected with *Lg* M4147 LRV^high^ or *Lg* M4147 LRV^neg^, we tested for the presence of LRV. As shown in Figure 8, LRV was only detected in lesion extracts from *Lg* M4147 LRV^high^ but not in *Lg* M4147 LRV^neg^. Importantly, it was visible in as little as 25–50 ng of total RNA extract, which corresponds to a clinically minute biopsy size (approximately 20–50 µg of lesion). This result showed that, not only we can detect LRV in promastigotes but also in amastigotes in lesion lysates, suggesting that this detection method has great potential for use in the field.

## Discussion

The presence of LRV in *Leishmania (Viannia)* species is suspected to be a major aggravating factor in the dissemination and persistence of leishmaniasis. Therefore, the detection of LRV might prove clinically beneficial, guiding treatment or providing prognostic information. In this study, we evaluated several approaches of LRV detection, starting with the identification of a 5.3 kb viral dsRNA band in total parasitic nucleic acid (Figure 1A) [14]. This method, however, had the marked disadvantage of requiring at least 10^8^ parasites and a high LRV load. On the contrary, the qRT-PCR approach is both highly sensitive as well as quantitative but its use as a first line diagnostic could be limited in the field in case of LRV genetic polymorphism (as illustrated with the SetB primers in Figure 1C). Further work would be required to identify the highly conserved regions amongst all LRV genomes in divergent parasite strains in order to avoid such a problem. Immunodetection by anti-LRV antibodies also proved to be clinically applicable with the advantage of qualitative analysis by fluorescence microscopy, revealing an interesting cytosolic clustering of viral particles (Figure 2B). Anti-capsid antibodies, however, have the same potential limitation as qRT-PCR due to their dependence on the underlying capsid sequence.

In this report, we describe new sequence-independent LRV detection techniques, using the anti-dsRNA J2 antibody, which could then be used in the field against any LRV strain circumventing the problem of sequence specificity that could occur. It was found to be effective and quantitative in microscopy, slot blot, ELISA and dot blot assays using parasites or lesions extracts, where it detected LRV in all LRV-positive control strains. All the strains analyzed in this study and the results obtained from each method are summarized in Table 1. The anti-dsRNA-based dot blot technique stood out as the candidate method for use in the field, having sufficient sensitivity and ease of use to allow rapid LRV detection at a relatively low cost that could be performed at a large scale in a clinical setting (Table 1).

**Table 1:** LRV status of the analyzed strains according to detection method.

		dsRNA		WB + IFM	IFM	SB	ELISA	DB
LRV load	Strain	extraction	qRT-PCR	(capsid Ab)	(J2)	(J2)	(J2)	(J2)
**High**	*Lg* M4147 LRV^high^	**+** a	**+** a	**+**	**+**	**+**	**+**	**+**
	*Lg* 1398	**+** a	**+** § a	**−**	**+**		**+**	**+**
	*Lg* M5313	**+** * a ^,^ b	**+** * a	**+**	**+** *		**+** *	**+** *
	*Lg* 13	**+** * a	**+** * a	**+** *	**+** *			**+** *
	*Lg* 21	**+** * a	**+** * a	**+** *	**+** *			**+** *
	*Lb* 1064	**+** * b						**+**
	*Lb* 1174	**+** * b						**+**
	*Lb* 1403	**+** * b						**+**
	*Lb* 1407	**+** * b			**+**			**+**
	*Lb* 2169	**+**			**+** *			**+**
	*Lb* 2192							**+**
**Low**	*Lg* 1881	**−** a	**−** a	**−**	**−**		**−**	**−**
	*Lg* 03	**−** * a	**−** * a	**−** *	**−** *			**−** *
	*Lg* 17	**−** * a	**−** * a	**−**	**−** *		**−** *	**−** *
**Negative**	*Lg* M4147 LRV^neg^	**−** a	**−** a	**−**	**−**	**−**	**−**	**−**

a As shown in Ives *et al.*, 2011; by qRT-PCR analysis, *Lg* 1881, *Lg* 03 and *Lg* 17 were classified as LRV^low^ harboring at least 10,000 fold less viral transcripts than the highly infected strains.

b As shown in Salinas *et al.*, 1996.

* Performed in this study but not shown in the figures.

§ Only with specific primers.

Abbreviations: WB = western blotting/IFM = immunofluorescence microscopy/SB = slot blot/DB = dot blot.

In our previous analysis [14], we showed that the metastatic parasites in the Golden hamster model as well as a human ML isolate were positive for LRV, while non-metastatic and a human CL-derived strain were negative or very poorly infected. From the analysis reported here, we could detect the presence of LRV in other *Leishmania* isolates, including again *L. guyanensis*, but in addition in freshly isolated *L. braziliensis* parasites from human lesions. Finally, we showed that LRV could also be detected directly from minute lesion biopsies in mice thus avoiding parasite isolation and promastigote cultivation, which is a clear advantage when adapting of the technique such a diagnostic technique for field applicability. We propose that this approach could now be finalized for use on a mass-scale to determine the prevalence of LRV in *L. (Viannia)*. This would greatly aid in confirming the correlation between LRV presence and clinical phenotype. If a significant trend is established, LRV detection could be used as a prognostic tool, perhaps guiding treatment strategies to prevent the metastatic complications often observed in some *Leishmania (Viannia)* infected patients.
